# Clinical Manifestations and Diagnostic Challenges in Acute Porphyrias

**DOI:** 10.1155/2013/628602

**Published:** 2013-02-12

**Authors:** Henry Trier, Vikram P. Krishnasamy, Pashtoon Murtaza Kasi

**Affiliations:** ^1^Department of Internal Medicine, University of Pittsburgh Medical Center (UPMC), Pittsburgh, PA 15213, USA; ^2^International Scholars Program, Department of Internal Medicine, University of Pittsburgh Medical Center (UPMC), Pittsburgh, PA 15213, USA

## Abstract

The porphyrias are a group of disorders characterized by an enzyme deficiency in the heme biosynthetic pathway. These can be classified into either erythropoietic or hepatic forms depending on the site of the major enzyme deficiency. The diagnosis of acute porphyrias, however, can be very challenging due to overlapping features amongst the various types. Initial suspicion is based on a myriad of clinical manifestations, which then are confirmed by laboratory testing where available. Genetic testing is now also available for the different types of porphyrias, aiding in the definitive diagnosis. Here, we present a challenging case of porphyria in a patient with end-stage renal disease and present the diagnostic challenges associated with the case and the ways forward.

## 1. Case Presentation

A 34-year-old gentleman with a history of end stage renal disease (ESRD) secondary to focal segmental glomerulosclerosis, for which he is on hemodialysis, and unexplained peripheral neuropathy, presented to the hospital with severe intractable epigastric pain and dark urine.

Of note, the patient had two recent hospital admissions. The first admission, about a month prior, was due to a significant transaminitis (initial AST and ALT levels were 2851 IU/L and 3850 IU/L, resp.). Testing done at admission was notable for an elevated ferritin (>1500 ng/mL; subsequent genetic testing for the HFE gene was negative), negative viral hepatitis serologies, and negative tests for alpha 1 antitrypsin, ANA, antismooth muscle antibody, and antimitochondrial antibody. By discharge the AST and ALT had declined to 1446 IU/L and 298 IU/L, respectively. The liver injury was attributed to congestive hepatopathy secondary to missed dialysis sessions. The second admission, about a week prior, was due to altered mental status, hyperkalemia, epigastric abdominal pain, and a mild transaminitis (his ALT and AST had normalized in the interim) in the setting of missed dialysis sessions. His confusion and electrolyte abnormalities resolved with emergent and repeated dialysis. Of interest, during the second admission, the patient developed distal bullae on his feet.

On presentation, the patient endorsed numerous attacks of sharp, constant epigastric pain, which were not affected by food and were associated with both nausea and vomiting. He denied any changes in his bowel movements. This abdominal pain had an extensive prior workup. An esophagogastroduodenoscopy (EGD) done a week prior showed LA Grade A esophagitis, healing esophageal ulcers, mild gastritis, antral erosions, mild-to-moderate duodenitis, and one small duodenal ulcer with a clean base (*H. pylori* testing was negative). A right-upper quadrant ultrasound a week prior was without gallstones or biliary duct dilatation. An abdominal computerized tomography scan a month prior was unremarkable save hepatic steatosis. A normal gastric emptying study and hepatobiliary iminodiacetic acid (HIDA) scan were done seven months prior. For the imaged esophageal, gastric, and duodenal inflammation, the patient's medications were optimized to twice daily pantoprazole without subsequent alleviation of pain. The patient also endorsed one episode of “grape-juice-colored” urine the day prior to admission in the context of dysuria, without concurrent fever or chills.

**Figure 1:**
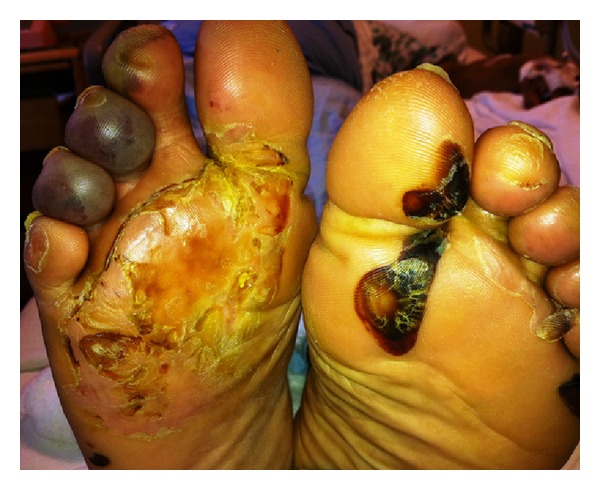
Blistering lesions in various stages of healing seen in our patient as noted in neurocutaneous porphyrias and porphyria cutanea tarda [5].

**Figure 2:**
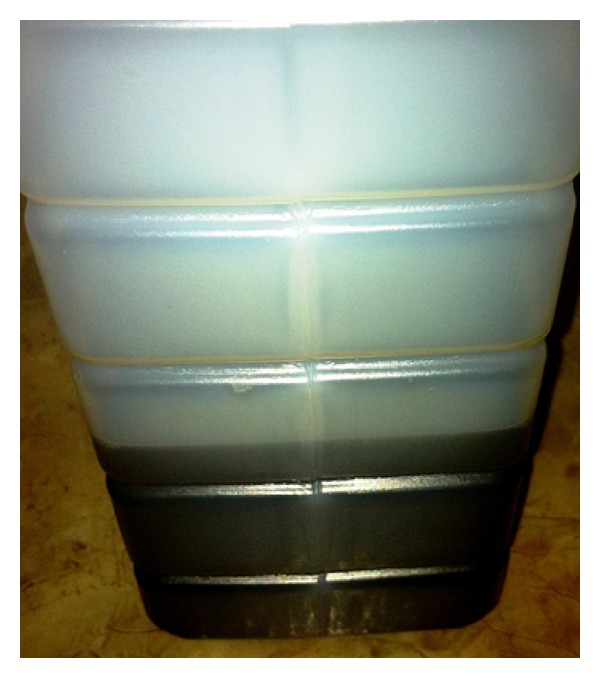
Dark urine noted during several of admissions for probable porphyria (urine may appear dark or purple during an attack or after standing in light due to the presence of porphyrins) [6].

The physical exam was notable for both epigastric and suprapubic tenderness, as well as a four-centimeter ulceration on the plantar aspect of his right foot, with several small ulcerations noted on the second and third digits of his left foot (Figure 1). His urine was noted to be purple to black in color (Figure 2). There was neither hyperpigmentation nor hypertrichosis. A urinalysis was done which was significant for 4+ blood, trace leukocyte esterase, negative nitrite, 478 WBCs, and 297 RBCs (Figure 2). WBC count was elevated at 12.6. For presumed prostatitis, the patient was started initially on IV ceftriaxone that was later transitioned to a course of oral ciprofloxacin. Repeat urinalysis done 2 days later was significant for 2+ blood, negative leukocyte esterase, negative nitrite, 3 WBCs, and no RBCs. WBC count decreased to 6.6 during this period.

The patient on routine labs was also found to have an acute on chronic anemia (hemoglobin baseline 10-11 g/dL, Hgb on admission 9.7 g/dL with an MCV of 92.6 and an RDW of 19.9). Further evaluation was consistent with a hemolytic anemia (Table 2). Subsequent workup for an etiology of the hemolysis was negative including hemoglobin electrophoresis, Coombs test, and G6PD testing.

After review of the prior records depicting the recurrent presentations for abdominal pain (~30 emergency room visits), combined with the new onset of dark urine, the development of distal bullae on his feet, the unexplained past history of neuropathy, and the altered mental status with some ongoing psychiatric symptoms, a unifying diagnosis of possible acute intermittent porphyria was considered and urine and serum porphyria labs were sent.

Following is an account of the clinical manifestations and the challenges faced in the diagnosis of acute porphyrias along with the discussion of the case. 

## 2. Discussion

The porphyrias are a group of disorders characterized by an enzyme deficiency in the heme biosynthetic pathway. These can be classified into either erythropoietic or hepatic forms depending on the site of the major enzyme deficiency, but often, this task is complicated by overlapping features. From the clinical standpoint, they can be also divided into acute and nonacute forms depending on the presence or absence of an acute porphyria attack [7, 10]. Initial suspicion of a possible diagnosis is based on a myriad of clinical manifestations (Table 1), which appear to have significant variance and are confirmed by laboratory testing where available [11]. 

The most common acute porphyria is acute intermittent porphyria (AIP), an autosomal dominant disorder of an enzyme called porphobilinogen (PBG) deaminase [12]. The disease is characterized by a partial deficiency of this enzyme (half normal activity), and thus the symptoms of AIP may not appear until the second or third decade of life. Exposure to a precipitating agent can lead to an earlier presentation [13]. Women are more commonly affected than men. Hormonal factors have been implicated, giving explanation to why AIP may not be apparent until puberty [14]. Penetrance is also variable, so a family history may not be present in some cases. 

AIP is one of the acute hepatic porphyrias, a group that also includes hereditary coproporphyria (HCP), variegate porphyria (VP), and ALA dehydratase deficient porphyria (ALAD). These classically present with a neurological complaint [15, 16]. Peripheral neuropathy is the most common central nervous system manifestation. Motor symptoms tend to affect the proximal muscles, more frequently the upper extremities. Sensory symptoms are less common than motor, but include paresthesias and sensory loss. The autonomic nervous system can also be affected leading to tachycardia, hypertension, tremor, and sweats. 

Of the acute hepatic porphyrias, cutaneous manifestations in sun exposed areas are usually seen in VP and HCP. Thus, these two are sometimes referred to as the neurocutaneous porphyrias. The dermatologic manifestations are similar to the bullous lesions seen with porphyria cutanea tarda (PCT). VP is common in some parts of the world including South Africa. HCP, however, is extremely rare. 

Diagnosis of an acute porphyria attack is usually suggested by a triad of symptoms: visceral abdominal pain, neurological dysfunction, and psychiatric disturbances, such as mental status changes. Of these, abdominal pain is the most troublesome for patients and is the most frequent cause of hospital admission [17]. This trend is exemplified by our patient who had more than 30 emergency room visits within the past year for abdominal pain. Since the severity of abdominal pain in an acute porphyria attack can mimic that of an acute abdomen, the acute hepatic porphyrias are of interest for a broad group of specialists, including internists, gastroenterologists, surgeons, and gynecologists [7]. Abdominal pain occurs in 85–95% of patients. It can be constant with poor localization and is often accompanied by nausea, vomiting, constipation, and ileus. Since symptoms are more neuropathic than inflammatory, leukocytosis and fever are generally not present during an acute attack. Over time, repeated attacks can lead to chronic pain and elevated transaminases. Bladder dysfunction may also be present, accompanied by changes in urine color, which is usually secondary to a hemolysis noted in some porphyrias.

Psychiatric manifestations accompany acute attacks in up to 80% of the cases. Symptoms include anxiety, depression, hallucinations, paranoia, and mental status changes (ranging from mild confusion to encephalopathy and coma). Our patient had multiple admissions for altered mental status and encephalopathy of unknown etiology. 

In addition, both hypertension and a chronic kidney disease are reported as consequences of long-term symptomatic disease in acute porphyrias [1]. Worsening renal insufficiency can increase both the severity and frequency of porphyria attacks [2]. Of note, our patient had ESRD, which was poorly controlled given multiple missed dialysis sessions.

Due to the high degree of symptom variance, it is crucial to have a high index of suspicion when making the diagnosis. Initial testing for AIP involves measurement of urinary porphobilinogen (PBG). Levels tend to be normal during asymptomatic periods but are significantly increased in both the urine and blood during acute attacks, thus making it paramount to draw labs during an acute attack. Urine levels during acute attacks are generally 50–200 mg/day. However, elevated levels can also be seen with HCP or VP. If initial testing is negative on a spot urine sample but clinical suspicion remains high, a 24-hour urine collection along with quantitative measurements of delta-aminolevulinic acid (ALA), PBG, and total porphyrins should be obtained. 

This workup can be challenging in patients with end-stage renal disease (as in our case) and in patients who are anuric. In these patients, plasma measurements are of particular importance with the reference range for the values being higher in patients with renal failure than in normal individuals [18]. Hindmarsh and colleagues have reported on the plasma profiles observed in those with porphyria and those with severe renal failure with skin lesions typical for PCT (a process coined pseudoporphyria of renal failure) to help determine patterns and reference ranges for this particular subset of patients where interpretation of the lab values can be particularly difficult [19]. 

Once elevated levels have been identified, one must still differentiate AIP from other porphyrias such as HCP and VP. To answer this question, measurements of erythrocyte PBG deaminase (PBGD) activity along with quantitative measurements of porphyrins in the plasma, urine, and feces need to be obtained. Low erythrocyte PBG activity helps to verify the diagnosis of AIP but is not an effective tool for initial diagnosis. Plasma porphyrins are elevated in VP, and fecal porphyrins, while normal in AIP, tend to be significantly elevated in both HCP and VP. Some of the fecal porphyrins can even remain elevated between periods of attacks. It is therefore extremely important to have complete biochemical profiles of plasma, urine, and fecal porphyrins available. Expert consultation here may be of great value in helping to distinguish amongst the porphyrias. Unfortunately, with respect to our patient, the plasma levels of some of the labs were collected initially, but the urine labs were not collected until two weeks later. The spectrum of his lab abnormalities is outlined in Table 2. 

Based on the data obtained, our patient exhibits the clinical profile of an acute hepatic porphyria (the neurocutaneous porphyrias VP and HCP more so than AIP). However, his biochemical profile is confusing and overlaps with CEP, HEP, VP, HCP, and PCT. Of note, concurrent conditions like a hemolytic anemia may lead to increased premature cells in the circulation, leading to a falsely normal enzyme activity level (used to screen for porphyrias). In addition, interpretation of these profiles in the setting of ESRD is particularly difficult. The pseudoporphyrias of renal failure and bullous lesions seen in some patients with ESRD are other items to consider in the differential diagnosis.

Our patient had an episode of severe liver injury which preceded the onset of both the severe epigastric pain and the bullous lesions. This course raises the possibility that given the hepatic synthesis of the enzymes involved, the prior insult may have tipped him over to the current presentation. Another interesting fact is the association of PCT with conditions predisposing to iron overload (hemochromatosis and ESRD) and increased hepatic deposition of iron as a precipitating factor. 

Genetic testing greatly helps to establish a diagnosis when the clinical presentation and the laboratory results may not exactly fit one type of porphyria. Currently, DNA testing is not readily available at most centers. The Mount Sinai Genetic Testing Laboratory in New York City does DNA testing for seven porphyrias: AIP, HCP, VP, familial PCT, hepatoerythropoietic porphyria (HEP), erythropoietic protoporphyria (EPP), and congenital erythropoietic porphyria (CEP). Apparently, this is the only laboratory in the United States that offers DNA testing for all of these porphyrias. The estimated cost for DNA testing as reported on the American Porphyria Foundation's website for one specific porphyria is approximately $850. Three porphyrias can be tested for approximately $1850. Results from DNA testing are typically available in 2 to 4 weeks [6]. Unfortunately, our patient did not want to pursue the diagnosis, refused genetic testing, and was lost to followup.

Once the diagnosis is made, patient education regarding prevention is a key. Acute attacks are often precipitated by a number of factors, most commonly alcohol, low-calorie diets, hormones, and numerous drugs (an updated database of unsafe medications can be found at http://www.porphyriafoundation.com). Just providing patients with information about precipitants and the importance of avoiding them can decrease the number of patients who present with acute attacks by 80–90%, and half will have milder symptoms during their lifetime [3].

## Figures and Tables

**Table 1:** Clinical manifestations seen in attacks of acute porphyrias and findings noted in our case [1–4].

Findings seen in patients with porphyria	Findings seen in our patient
Neuropsychiatric manifestations	

Autonomic nervous system (tachycardia, arrhythmias, restlessness, tremor, sweating, etc.)	Tachycardia noted on some hospital admissions, but at the same time, the patient is on a beta blocker.
Peripheral	
Sensory	
Neuropathy (peripheral sensory)	Has underlying neuropathy without any other underlying cause, on gabapentin.
Motor	
Motor paresis	
Central nervous system	
Impairment of bulbar or respiratory function (respiratory paralysis)	
Convulsions/seizures	
Psychiatric	
Psychiatric manifestations (behavior change, agitation, anxiety, and depression)	Has ongoing anxiety/depression along with some agitation episodes.
Mental status changes	Multiple admissions for mental status changes.

Visceral manifestations	

(i) Abdominal pain (ii) Other locations of pain (chest, back, and limb)	More than 30 presentations over the past couple of years for abdominal pain and some for chest pain; has necessitated at least 6 computerized tomography (CT scans) and multiple ultrasound examinations.
*Other GI symptoms: constipation, ileus, vomiting, *and *abdominal distention *	Present in our patient.
Bladder dysfunction (urinary retention, incontinence, and dysuria)	Endorsed dysuria on some admissions.
*HTN *	Noted in our patient.
*Chronic kidney disease *	Present, necessitating dialysis.
Hyponatremia (from syndrome of inappropriate ADH secretion (SIADH))	Hyponatremia noted on some admissions but exact etiology not worked up.
Dark-colored urine	See Figure 2.

Cutaneous manifestations	

Bullous lesions usually uncommon in AIP (except for some patients with ESRD) but seen more so in neurocutaneous porphyrias (VP and HCP). Lesions not distinguishable from those of PCT.	See Figure 1.

**Table 2:** Biochemical abnormalities noted in our patient [1, 7–9].

Labs	Level				Normal range	Usually consistent with^++^
Plasma						
Porphobilinogen deaminase (PBGD activity erythrocyte)^2^			16.1		<6.0 nmol/L/s (diminished)	Normal in HCP and VP, and usually deficient in AIP.
Total porphyrins (plasma)^3^	22.6	↑	8.2	↑	1.0–5.6 mcg/L	
** ** *Coproporphyrins *	*7.3 *	↑	*1.8 *	↑	*≤0.8 *mcg/L	*HCP, VP,* and CEP.
** ** *Heptacarboxyporphyrins *	*1.4 *	↑	*0.6 *	↑	*≤0.2 *mcg/L	PCT and HEP.
Hexacarboxyporphyrins	ND		ND		≤0.3 mcg/L	
** ** *Pentacarboxyporphyrins *	*0.9 *	↑	*ND *		*≤0.4 *mcg/L	PCT, *VP. *
* Protoporphyrins *	*8.7 *	↑	*2.8 *		*0.4–4.8 *mcg/L	EPP, *VP. *
* Uroporphyrins *	*4.3 *	↑	*3.0 *	↑	*≤0.2 *mcg/L	PCT, *VP. *
Zinc protoporphyrin (erythrocyte)			108	↑	<100 mcg/dL	HEP mainly, but nonspecific increases common in other types of porphyrias including ADP.
Urine						
Delta-aminolevulinic acid^1^	0.3		0.5		<1.8 mg/g creat	*HCP, VP,* CEP, EPP, HEP, and PCT.
24-hour urine labs^3^						
Total porphyrins			*55.2 *		*31–139 *mg/g* creat *	
Coproporphyrins			*10.7 *	↓	*23–130 *mg/g* creat *	
Heptaporphyrin			*3.2 *		*≤4.6 *mg/g* creat *	
Hexaporphyrins			*ND *		*Undetectable *	
Pentaporphyrin			*ND *		*≤1.7 *mg/g* creat *	
*Uroporphyrin *			*41.3 *	↑	*≤22 *mg/g* creat *	*VP, CEP, HEP,* and *PCT. *
Fecal profile (not available)						
Other labs						
Ferritin	>1500				10–282 ng/mL	Iron overload precipitates PCT.
Iron sats	91–125%				25–50%	
LDH	2512	↑	2070	↑	<171 IU/L	Hemolytic anemia usually most severe in CEP, but can be seen to some extent with other porphyrias.
Haptoglobin	<5.8	↓	<5.8	↓	36–195 mg/dL	

^++^Based on the literature, some of the patterns noted were not consistently present in other studies, and overlapping trends have been reported, further signifying the need for genetic diagnosis in such cases.

^1^Delta-aminolevulinic Acid: +ve (acute intermittent porphyria, ALA dehydratase deficiency), −ve (congenital erythropoietic porphyria, erythropoietic protoporphyria, hepatoerythropoietic porphyria, and porphyria cutanea tarda), and +/− (hereditary coproporphyria, variegate porphyria).

^2^Please note that 5–10% of affected individuals have normal PBGD activity in erythrocytes.

^3^Patients with hereditary forms of porphyria usually will present with profound elevations of these analytes (>5-fold) during acute episodes. Moderate elevations (<3-fold) are more often due to medications or environmental factors.
